# The Tuning of LIPSS Wettability during Laser Machining and through Post-Processing

**DOI:** 10.3390/nano11040973

**Published:** 2021-04-10

**Authors:** Michael J. Wood, Phillip Servio, Anne-Marie Kietzig

**Affiliations:** Department of Chemical Engineering, McGill University, Montréal, QC H3A 0C5, Canada; michael.wood3@mail.mcgill.ca (M.J.W.); phillip.servio@mcgill.ca (P.S.)

**Keywords:** fs-laser, micromachining, wetting, hydrophobicity, LIPSS

## Abstract

In this work, we investigate the fabrication of stainless-steel substrates decorated with laser-induced periodic surface structures (LIPSS) of both hydrophilic and hydrophobic wettability through different post-processing manipulation. In carrying out these experiments, we have found that while a CO_2_-rich atmosphere during irradiation does not affect final wettability, residence in such an atmosphere after irradiation does indeed increase hydrophobicity. Contrarily, residence in a boiling water bath will instead lead to a hydrophilic surface. Further, our experiments show the importance of removing non-sintered nanoparticles and agglomerates after laser micromachining. If they are not removed, we demonstrate that the nanoparticle agglomerates themselves become hydrophobic, creating a Cassie air-trapping layer on the surface which presents with water contact angles of 180°. However, such a surface lacks robustness; the particles are removed with the contacting water. What is left behind are LIPSS which are integral to the surface and have largely been blocked from reacting with the surrounding atmosphere. The actual surface presents with a water contact angle of approximately 80°. Finally, we show that chemical reactions on these metallic surfaces decorated with only LIPSS are comparatively slower than the reactions on metals irradiated to have hierarchical roughness. This is shown to be an important consideration to achieve the highest degree of hydro-philicity/phobicity possible. For example, repeated contact with water from goniometric measurements over the first 30 days following laser micromachining is shown to reduce the ultimate wettability of the surface to approximately 65°, compared to 135° when the surface is left undisturbed for 30 days.

## 1. Introduction

The irradiation of solids with linearly-polarized laser radiation has long been known to result in the formation of laser-induced periodic surface structures (LIPSS) [1]. Because of the contactless, maskless, single-step nature of laser irradiation—coupled with advances in high repetition rate, low pulse duration laser technology—the processing of LIPSS has become a scalable method of surface functionalization [2]. Specifically, the wettability of a surface can be controlled by combining the regular nanoscale roughness imparted through laser irradiation with favourable surface chemistry. A highly hydrophilic surface, for example, can be fabricated through the creation of an increased hydrophilic surface area—leading to the Wenzel wetting state [3]. Contrarily, a highly hydrophobic surface can be fabricated through the creation of trapped air pockets—leading to the Cassie wetting state [4].

The typical method of rendering laser-irradiated solids highly hydrophobic had been to deposit a low-energy coating onto the micromachined surface [5]. However, Kietzig et al. (2009) reported the creation of superhydrophobic surfaces from smooth, hydrophilic, steel alloys some time after laser irradiation without the need for such chemical coatings. They noted that the water contact angle measured on their micromachined samples evolved from hydrophilic to highly hydrophobic over the first 2–3 weeks following exposure to the femtosecond laser beam, with the samples exposed simply to lab air. It was noted via surface chemistry analyses that the increase in hydrophobicity correlated with an increase in the relative amount of carbon—with the surface being solely made up of iron oxides and carbon after some time [6]. Kietzig et al. (2011) performed a follow-up study in which they showed that pure metals exhibit this same hydrophilic-to-highly hydrophobic wetting evolution upon laser irradiation, similarly following an increase in surface carbon content [7]. Indeed, in the years since these results were published, there have been a considerable number of studies carried out which demonstrate similar wetting evolutions for laser micromachined metallic surfaces, generating much interest around the ability to create superhydrophobic metals in a single processing step [8,9,10,11,12,13,14].

A specific mechanism or reaction scheme for the increase in surface carbon content after irradiating metals with ultrashort laser pulses remains unknown. However, it is clear that a sort of cleaning of the metallic surface occurs during the ablation process whereas the outermost oxidized layers are removed, exposing a hydrophilic reactive surface on which a new chemical layer can form [15]. Kietzig et al. (2009) presented their original hypothesis that the progressive dissociative adsorption of CO_2_ onto the reactive surface was the cause of the increase in non-polar carbon—and increased hydrophobicity. They tested this hypothesis by exposing a freshly irradiated surface to a CO_2_-rich environment. Indeed, this exposure led to a surface with a higher carbon content and a higher water contact angle than a surface exposed to lab air [6]. This technique of exposing a micromachined metal to a CO_2_-rich environment has since been reported as an experimental method to fabricate superhydrophobic surfaces [16,17]. On the contrary, Long et al. (2015) were the first to report that exposure of a laser-irradiated metal to pure CO_2_ actually led to a restraint in the hydrophilic-to-highly hydrophobic wetting transition. Rather, they report that residence in a vacuum atmosphere accelerated the transition due to adsorption of organic matter, originating from the mechanical vacuum pump, which had contaminated the chamber. Their theory of the progressive adsorption of hydrocarbons being the origin of the non-polar carbon surface chemistry was tested by exposing a laser-irradiated sample to an atmosphere rich in organic matter, which again led to accelerated transitions [10]. This contrary theory that airborne hydrocarbons are the source of the non-polar carbon which progressively adsorbs onto laser-irradiated metals—rendering them highly hydrophobic—has been the basis of discussion in many subsequent studies [12,13,15,18,19].

The creation of a stable highly hydrophilic metallic surface upon laser micromachining had also been reported by Kietzig et al. (2009). Instead of exposing the freshly irradiated surface to lab air or CO_2_, it was immersed in boiling water for two days. This residence in the water bath led to a surface chemistry rich in hydrophilic oxides, hypothesized to be formed through dissociative adsorption of the water molecules. This surface did not undergo the typical hydrophilic-to-highly hydrophobic wetting transition after exposure to lab air [6].

The manipulation of the micromachined substrate in the early times after irradiation is evidently paramount to the final wettability obtained. This has led to interest into the effect of the atmosphere during the irradiation process itself on final wettability of the surface. Pou et al. (2019) performed a study in which they streamed: air, Ar, N_2_, O_2_, and CO_2_ onto the substrate during nanosecond laser irradiation. After irradiation, the samples were individually sealed in an air-filled bag for a week before analytical tests were performed. What they report is that streaming N_2_, O_2_, CO_2_, or even simply streaming air during the irradiation process ultimately results in a hydrophilic surface rich in metal lattice oxides and hydroxides [20]. The origins of the air used in these experiments is not explicitly stated, thus it is difficult to determine if these results fit with the theory that airborne hydrocarbons are the source of the non-polar carbon which progressively adsorbs onto laser-irradiated metals. Further, the negative effect that the CO_2_ stream has on ultimate wettability of the surface contradicts what has been reported by those already mentioned.

Pou et al. (2019) make a point of mentioning in their experimental section that after their texturing process, the samples were rinsed with ethanol to remove any debris that could affect subsequent measurements [20]. This is an important procedural point considering that the waste generated during laser micromachining are reactive nanoparticles. Analytical techniques such as water contact angle or surface chemistry measurements could be greatly influenced by the presence of non-sintered nanoparticles or agglomerates. Nonetheless, it is often unclear how these nanoparticles are dealt with in published studies—or if they are considered at all [7,8,11,12,13,14,15].

The present work takes another look at the effect of CO_2_ exposure on the final wettability of laser micromachined metals. Specifically, we explore the effect of this gas on the wettability of LIPSS-decorated stainless-steel in an attempt to rigorously describe what we have empirically known—that CO_2_ exposure does in fact promote hydrophobicity of our laser micromachined metals. We also take this opportunity to discuss otherwise how the manipulation of LIPSS-decorated stainless-steel at early times following irradiation greatly affects the final wettability of the surface. In doing so, we note the effect of non-sintered nanoparticles generated during micromachining and the effect of disturbance of the nascent surface chemistry on the ultimate wettability. These are all important considerations as metal laser micromachining technologies get scaled and transferred to industrial applications.

## 2. Materials and Methods

### 2.1. Fs-Laser Micromachining

The substrate used in this study was a 0.036″-thick 316 stainless-steel sheet (McMaster-Carr). The manufacturer has supplied us with an ASTM A751/14a chemical analysis of the substrate, as presented in Table 1. This material came pre-polished with a mirror-like ASTM #8 finish. This sheet was cut into small coupons, and was then ultrasonically cleaned in acetone for 5 min to remove any oils from the surface prior to micromachining.

**Figure 1:**
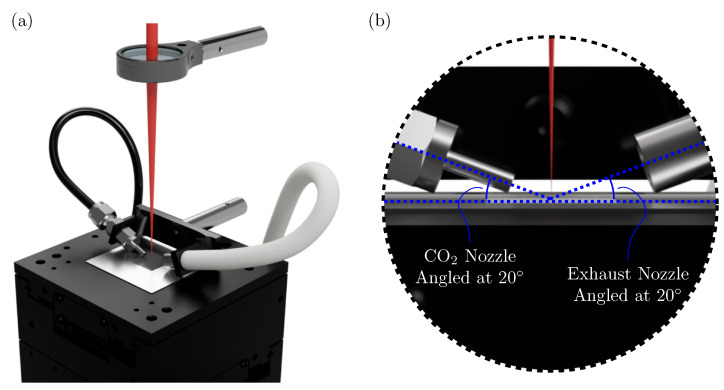
(**a**) Rendering of the fs-laser micromachining setup. Stainless-steel coupons are mounted horizontally on XY translation stages, 3 mm closer to the laser focusing lens than the focal point. A stationary nozzle is used to introduce CO_2_ gas at the point of laser irradiation. (**b**) Magnified rendering of the CO_2_ nozzle and exhaust line in relation to the stainless-steel substrate and laser beam spot.

The cleaned surfaces were in turn decorated with laser-induced periodic surface structures (LIPSS) by exposure to ultrashort laser pulses. The laser system used was a *Libra* amplified Ti:sapphire laser (Coherent, Inc., Santa Clara, CA, USA) with a central wavelength of 800 nm, output power of 4 W, pulse duration of <100 fs, and a 1 kHz repetition rate. A variable attenuator comprised of a half-wave plate and a polarizing beam splitter was used to reduce the laser output power. A plano-convex lens with a focal length of 200 mm was used to focus the laser beam. The pristine substrates were mounted horizontally onto XY linear translation stages, with their top surface being 3 mm closer to the focusing lens than the focal point, as shown in Figure 1a. The XY-stages were actuated by an *XPS* universal high-performance motion/driver controller (Newport Corp., Irvine, CA, USA). Successive pulses were overlapped in lines irradiated at 10 mm/s. Using the methods of Liu [21], the ablation threshold fluence of the 316 stainless-steel at these conditions was measured to be 0.23 J·cm^−2^, matching well what is reported in the literature [22]. Further, the effective beam diameter at these conditions is measured to be 80 µm. Through trial-and-error, optimal conditions for the formation of LIPSS were determined to be at a peak intensity of 696 W·cm^−2^, pulse fluence of 0.70 J·cm^−2^, and N_eff,2D_ of 200 pulses-per-spot. In total, a 10×10 mm square patch was irradiated on each stainless-steel coupon on which to perform the subsequent goniometric measurements.

A gas nozzle with an inner diameter of 2 mm was mounted stationary with the laser beam at an angle of 20° to the stainless-steel substrate, as shown in Figure 1b. This nozzle allowed flowing of carbon dioxide directly on the laser spot during irradiation, and thus a CO_2_-rich environment to be formed over the irradiated patch. A needle valve and calibrated in-line rotameter allowed for the carbon dioxide volumetric flow rate to be set as an experimental parameter (from 0 to 10,000 mL·min^−1^). An exhaust line was mounted opposite the CO_2_ nozzle as a safety measure to collect nanoparticles blown away by the gas stream.

### 2.2. Post-Processing Steps

The samples in the present work were prepared in three ways: (1) no further processing after laser micromachining—referred to hereafter as *no-post*; (2) residence in a CO_2_-rich reactor after micromachining—referred to hereafter as *CO_2_-reactor*; and (3) residence in boiling water after micromachining—referred to hereafter as *boiling-water*.

*CO_2_-reactor* samples were immediately removed from the XY translation stages after the micromachining routine had finished. They were placed in a small reactor with an ambient temperature of 60 °C, held constant via a PID-controlled heating element. Carbon dioxide was introduced to the reactor with its release valve initially open to flush the volume of air. The release valve was closed, the CO_2_ pressure inside the reactor was increased to 20 PSI, and the reactor was sealed. These samples had a residence time of 48 h inside the reactor before further tests were performed.

*Boiling-water* samples were immediately removed from the XY translation stages after the micromachining routine had finished. They were placed in a beaker of boiling deionized water which was continually topped off to maintain an adequate level of water. These samples had a residence time of 48 h in the boiling water before further tests were performed.

### 2.3. Sample Characterization

Carefully timed dynamic water contact angle measurements were performed on each of the micromachined stainless-steel coupons. These measurements were taken at 0-, 10-, 20-, and 30-days after micromachining was performed. The 0-day measurements were started within 10 min of the experimental surface being created (after finishing micromachining, or after finishing the post-processing step, as applicable). These water contact angle measurements were performed using a lab-built goniometer comprised of an *Infinity 3* microscope camera (Teledyne Lumenera, Inc., Ottawa, ON, Canada.) equipped with a *VZM 200i* zoom imaging lens (Edmund Optics, Inc., Barrington, NJ, USA). High backlighting is provided by an ultra-bright LED spotlight (Optikon Corp, Kitchener, ON, Canada). A 70-2203 syringe pump module (Harvard Apparatus, Inc., Holliston, MA, USA), controlled by a custom *Arduino* code, pumps degassed reverse osmosis water onto the studied surface. $\theta_{\mathrm{sessile}}$ was measured by gently placing a 5 µL droplet onto the studied surface. $\theta_{\mathrm{advancing}}$ and $\theta_{\mathrm{receding}}$ were measured by first gently placing a 2 µL droplet onto the studied surface, then pumping 5 µL to and from said droplet, respectively, at a rate of 0.25 µL/s.

The goniometric measurements on each stainless-steel coupon were carried out as follows. First, $\theta_{\mathrm{sessile}}$ was measured on five different locations within the laser-irradiated patch. In the case of *CO_2_-reactor* samples, a second round of $\theta_{\mathrm{sessile}}$ measurements was performed on the same five locations within the patch, after allowing the surface to dry. Next, $\theta_{\mathrm{advancing}}$ and $\theta_{\mathrm{receding}}$ were measured on the same five locations within the patch, after allowing the surface to dry. The error bars in the data presented herein represent the bounds of the 95% confidence intervals on the mean of these goniometric measurements.

After the 30-day goniometric measurements were completed all of the substrates described in the previous sections were imaged using a *Quanta 450* scanning electron microscope (ThermoFisher, Inc., Waltham, MA, USA). An accelerating voltage of 5.0 kV and spot size of 2.5 nm were used to image the stainless-steel samples.

## 3. Results and Discussion

### 3.1. Effect of CO_2_ Stream Flow Rate

**Figure 2:**
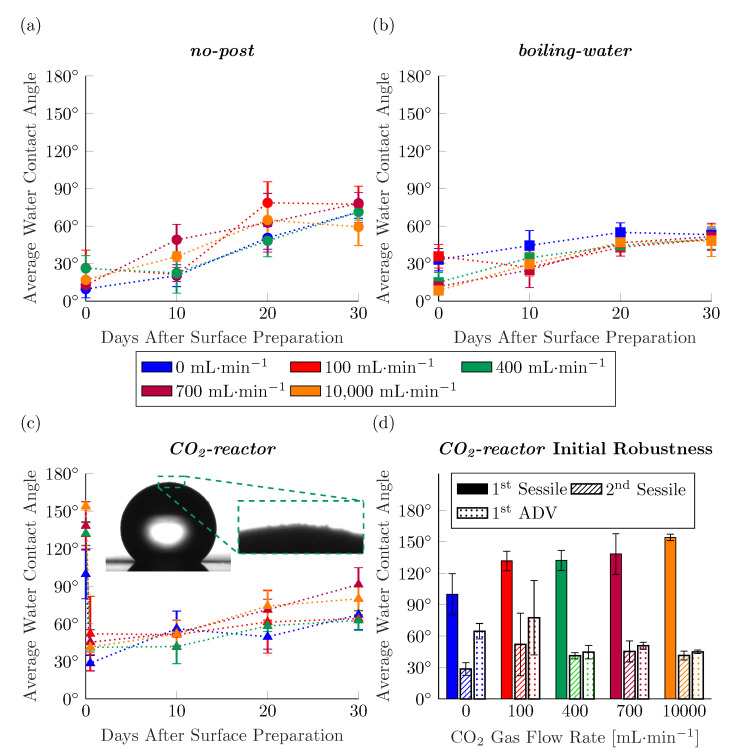
Average sessile water contact angle measurements taken over the 30 days following laser micromachining/post-processing on surfaces prepared with: (**a**) no post-processing step; (**b**) 48 h in boiling water; and (**c**) 48 h in the CO_2_ reactor. Inset shows nanoparticle agglomerates on periphery of first sessile drop placed on *CO_2_-reactor* surfaces. (**d**) Comparison of the average: contact angle of the first sessile drop, contact angle of the second sessile drop, and advancing contact angle at day 0 on *CO_2_-reactor* surfaces.

We begin the discussion with an overview presentation of the bulk of the data collected. Figure 2a–c presents the average sessile water contact angles measured over the 30-day period on the laser micromachined surfaces prepared with increasing flow rate of CO_2_ during irradiation, and the three separate post-processing steps. We note that the dynamic water contact angles are typically better suited to describe the wettability of a surface, and indeed we include this data as Supplementary Materials, however the nature of our experimental method leads us to centre our discussion around both datasets, as explained later.

The data in Figure 2a–c show that the flow rate of CO_2_ gas during fs-laser irradiation has no appreciable effect on the wettability of the resulting surface. There is no statistically-significant difference in contact angles measured on those surfaces prepared with no CO_2_ stream, and those surfaces prepared with increasing volumetric flow rates of CO_2_. This lack of effect persists over the 30 days of goniometric measurements following surface preparation. It is important to highlight that in this data we do not see a negative effect on wettability of the surface from streaming CO_2_ during irradiation, as was reported by Pou et al. [20]. A possible explanation for these results is that the rate of the reaction which re-forms the surface chemistry on the laser irradiated stainless-steel is far too slow to be affected by the CO_2_ surrounding the micromachining step itself. Very little is known about the kinetics of this chemical reaction, yet it is often reported that water contact angles continue to increase (and hence the surface chemistry continues to change) for around 30 days following laser irradiation [6,15].

An interesting result that was evident during experimentation was the exceptionally high sessile contact angles presented by the *CO_2_-reactor* surfaces immediately following their 48-hour residences (Figure 2c). Contrary to the rest of the data collected in the present work, the flow rate of CO_2_ during laser irradiation seemed to have a positive effect on the resulting contact angles. However, the first sessile droplets placed on these surfaces clearly had agglomerated nanoparticles at their periphery, as exemplified by the inset images of Figure 2c. Evidently, nanoparticles generated during laser micromachining agglomerate and remain, non-sintered, atop the laser-induced periodic surface surface structures. These agglomerates are hydrophobic following the residence in the CO_2_ reactor and lead to the air-trapping characteristic of the Cassie wetting state. The coverage of the LIPSS-decorated surfaces with nanoparticles and nanoparticle agglomerates is an important consideration both in the research context, and as laser-irradiated surface technology starts being transferred to industry. Observations such as the inset image of Figure 2c bring into question whence the positive characteristics of a laser-irradiated surface arrive. For in effect, the first sessile droplet placed on the *CO_2_-reactor* surfaces is measuring the hydrophobicity of the compound LIPSS–nanoparticle agglomerate system. As the surface is covered with more and more nanoparticle agglomerates, a sessile drop coming into contact with it will resemble more and more a Janus particle. Surfaces such as these inherently lack robustness, as rolling droplets of water carry away the nanoparticle agglomerates, leaving behind the underlying substrate.

To illustrate the lack of anti-wetting robustness, we placed a second sessile droplet at approximately the same locations on the *CO_2_-reactor* surfaces at Day 0, allowing the surface sufficient time to dry after the first sessile droplet. Figure 2d shows that while these surfaces present as hydrophobic (even highly-hydrophobic with increased CO_2_ flow rate during micromachining) with the first sessile droplet placed on the surface, they present as hydrophilic with the second sessile droplet. Further, the increased volumetric flow rate of the CO_2_ jet has not lead to a statistically-significant increase in the average contact angles of the second sessile droplet. This is further evidence that the rate of the reaction which re-forms the surface chemistry of the LIPSS-decorated stainless-steel is too slow to be affected by the composition of the atmosphere surrounding the micromachining process itself. Figure 2d also presents the average advancing contact angle measured at approximately the same locations as the first and second sessile droplets at Day 0. The data from these measurements are interesting in that they describe surfaces which are hydrophilic, however, in some cases the advancing contact angle is especially higher than the sessile contact angle previously measured and in some cases they are statistically-equivalent. The variability between the average contact angles measured by the second sessile droplets and first advancing droplets to touch the *CO_2_-reactor* surfaces likely stems from the exact location where these droplets were placed. That is, the wettability of the system depends greatly on the ratio of hydrophobic nanoparticle agglomerates to hydrophilic LIPSS.

**Figure 3:**
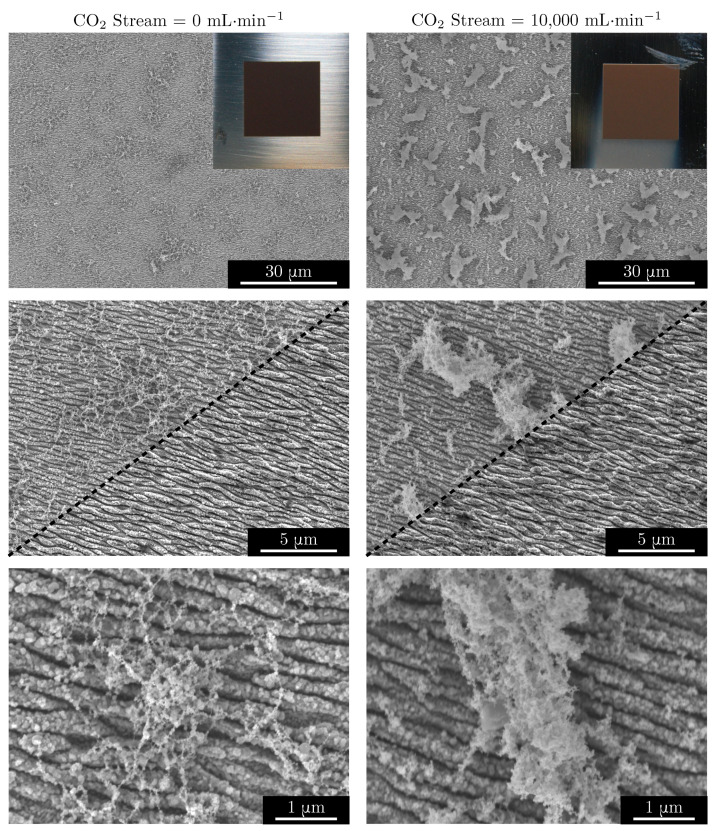
Scanning electron micrographs of the surfaces produced by fs-laser micromachining with (right column) and without (left column) a CO_2_ stream. Inset photographs of the top row demonstrate the CO_2_ jet serves to confine the area of nanoparticle (agglomerate) redeposition to downstream from the nozzle. Middle row shows a comparison between the surfaces after micromachining (upper left) and after ultrasonication (lower right).

To explore the non-sintered nanoparticle agglomerate phenomenon further, we micromachined two more LIPSS-decorated surfaces: one fabricated without CO_2_ streaming during micromachining and one fabricated with a 10,000 mL·min^−1^ stream of CO_2_. After micromachining, these samples were sputter-coated with 5 nm of platinum to afix any nanoparticles in place for scanning electron microscopy. The resulting scanning electron micrographs are presented in Figure 3.

The images in Figure 3 show clearly that nanoparticle agglomerates are formed and redeposited homogeneously on the surface during the fs-laser micromachining process, even at the low-fluence conditions necessary for the formation of only LIPSS. Micromachining with increased laser fluence—such as for the formation of bumpy self-assembled structures or inscribed geometric patterns—will only exacerbate this issue of nanoparticle agglomerate redeposition. The inset photographs in the top row of Figure 3 show the macroscopic results of streaming CO_2_ during irradiation. With no CO_2_ stream during micromachining, much of the perimeter surrounding the irradiated patch tends to become covered in a layer of non-sintered nanoparticles/agglomerates, giving the pristine stainless steel a golden brown hue. However, the streaming of CO_2_ gas during micromachining has confined the redeposition of nanoparticles and agglomerates to only downstream from the nozzle. Comparing the left and right columns of Figure 3, it is obvious that the jet of CO_2_ gas serves to increase the degree to which the underlying structured surface is covered with large nanoparticle agglomerates. As the flow rate of gas is increased, the expanding nanoparticle plume is confined to a greater degree and nanoparticles ejected from the substrate tend to agglomerate. These nanoparticle agglomerates are redirected back down to the surface where they homogeneously blanket the LIPSS. We theorize that the angle between the CO_2_ nozzle and the surface will also affect the degree of nanoparticle plume confinement. Whereas if the jet of CO_2_ is directed to the substrate from a higher incident angle, we would expect to see more coverage of the laser-irradiated patch with nanoparticle agglomerates, and therefore a positive effect on the initial hydrophobicity of the sample. The relationship between CO_2_ stream angle of incidence and initial surface hydrophobicity could be the topic of interesting future research.

The higher magnification micrographs of Figure 3 show that with increased CO_2_ flow rate, the nanoparticle agglomerates transition from spider web–like formations to large cloud–like flakes. That is, further confinement of the nanoparticle plume with higher stream flow rates means that more ejected hot nanoparticles come into contact and agglomerate. In addition to the large agglomerates, the LIPSS are left covered with a blanket of nanoparticles. The middle row of Figure 3 presents a side-by-side comparison of the surfaces before and after ultrasonication in acetone for 5 min. Clearly, ultrasonication has removed not only the large agglomerates from the irradiated patch, but also many of the nanoparticles—suggesting that they are redeposited without becoming sintered. Inspection of the micrographs from the ultrasonicated samples confirms that the stream of CO_2_—and the nanoparticle plume confinement which results—does not affect the appearance of LIPSS. In fact, as expected, both the surfaces prepared with no CO_2_ stream and a stream of 10,000 mL·min^−1^ possess the characteristic nanoripples with no appreciable difference.

**Figure 4:**
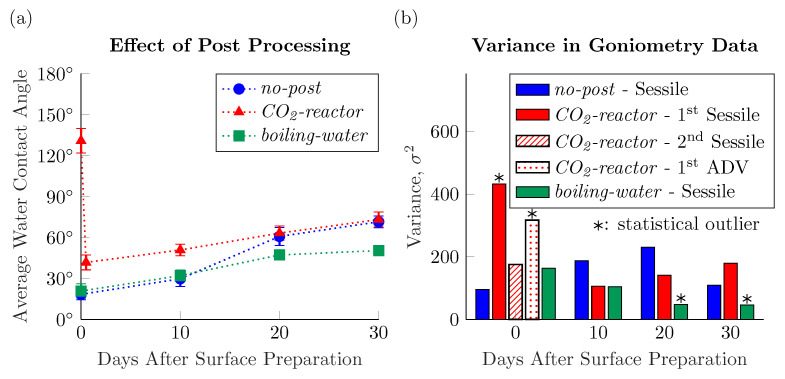
(**a**) Average sessile contact angle measured on the *no-post*, *CO_2_-reactor*, and *boiling-water* surfaces regardless of CO_2_ jet volumetric flow rate during micromachining over the 30 days following surface preparation. (**b**) Variance in the water contact angle data presented in part (**a**). Asterisk denotes a statistically-significant difference in variance compared to the rest of the dataset, as determined through F-tests.

As the volumetric flow rate of the CO_2_ streamed during the laser micromachining process was seen to have little effect on the hydrophobicity of the resulting surfaces—save for the initial hydrophobicity of *CO_2_-reactor* surfaces—we plot in Figure 4a the sessile contact angle data as an average of all surfaces prepared with the same post-processing step, regardless of stream flow rate. The very small 95% confidence intervals of the contact angle data presented this way is further evidence that the volumetric flow rate of the CO_2_ stream has no effect on the wettability of the resulting LIPSS-decorated surfaces. The variance in the data analyzed this way can also elucidate the homogeneity of the fabricated surfaces. Figure 4b presents the variance of each data point graphed in Figure 4a. In general the data of the present work is of low, consistent, variance with the exception of four outliers as determined by statistical F-tests. The two datasets with statistically *greater* variances are not surprisingly those contact angle measurements taken on the compound hydrophobic nanoparticle agglomerate—hydrophilic LIPSS systems. The variance in the contact angle measurements of the first sessile drops to touch the *CO_2_-reactor* surfaces is high because of the varying degree of nanoparticle agglomerate coverage with increasing CO_2_ stream flow rate. Thus, these surfaces manifest the Cassie wetting state to varying degrees, with more nanoparticle agglomerates leading to more of the air-trapping effect. The advancing contact angle data taken on the *CO_2_-reactor* surfaces on Day 0 also has statistically greater variability than those of the other datasets, backing up our hypothesis that as the footprint of the droplet is increased beyond the contact area of the original sessile droplets, it is in contact with a compound hydrophillic LIPSS–hydrophobic nanoparticle agglomerate system. What we find especially interesting in Figure 4b is that the contact angle data of the second sessile droplets to touch the *CO_2_-reactor* surfaces have a variance which matches well the variances of the bulk of the dataset. This low variability in the measured contact angles of the second sessile droplets to touch the *CO_2_-reactor* surfaces shows that the underlying micromachined substrate—left behind once the hydrophobic agglomerated nanoparticles are removed by the first sessile droplet—possesses homogeneous wettability, like the *no-post* and *boiling-water* surfaces.

The final two datasets presented in Figure 4 with statistically different variances are the measured sessile contact angles on the *boiling-water* surfaces 20 and 30 days after their initial preparation. Evidently, the residence in boiling water for 48 h has yielded surfaces with exceptionally homogeneous wettability. The high level of surface chemistry homogeneity could be the result of the washing away of any nanoparticle agglomerates which are on these surfaces after the micromachining step as well as a higher reaction rate for the formation of hydrophilic hydroxides in the water bath than for the formation of hydrophobic oxides in the CO_2_ chamber or lab air. This point is discussed further in the next section.

### 3.2. Effect of Water Contact

The temporal evolution of the studied surfaces’ wettabilities, as presented in Figure 4a, warrants further discussion. Kietzig et al. (2009), in their seminal work on the surface chemistry changes induced when metals are irradiated with ultrashort laser pulses, showed that hierarchically-rough fs-laser micromachined stainless-steels can be made to remain hydrophilic, in the Wenzel wetting state, if boiled in water for 2 days following the laser treatment [6]. We see similar, yet distinct results here for surfaces decorated with LIPSS alone. In Figure 4a, it is shown that the average sessile water contact angle measured on the *boiling-water* surfaces increases from 21±5° immediately after removal from the boiling water bath to 50±3° 30 days later. Whereas Kietzig et al. (2009) measured no appreciable change in water contact angle on their boiled surfaces, we see a nearly 30° increase. This suggests that the same 2-day residence in the boiling water bath was not sufficient to allow for the hot water to fully react with the laser irradiated surface to form a hydrophilic iron oxide layer. Rather, as the *boiling-water* surface sat exposed to lab air for 30 days, either some CO_2_ from the air dissociatively adsorbed to the surface [6] or airborne hydrocarbons adsorbed to the surface [15], forming some hydrophoboic carbonaceous compounds. The weakened reactivity of the surfaces in the present study—those decorated with only LIPSS—compared to the surfaces prepared by Kietzig et al. (2009) stems from the lessened fluence used to induce only the formation of the nanoripples. With lowered irradiation fluence comes a weakened cleaning effect where less of the outermost, more oxidized layers of the material are removed [15]. Therefore, there are less Fe(metal) and Fe(II) oxide components at the surface to evolve to more oxidized forms and hence the slower reaction rate [6,15]. Further, the single-scale roughness of the surfaces decorated with only LIPSS inherently possess less total reactive surface area than the hierarchically-rough surfaces created by the higher fluences employed by Kietzig et al.

Figure 4a shows that the wettability transition of the *no-post* surfaces do not match the typical exponential growths of those metals reported in the literature to be hierarchically-roughened by laser micromachining [6,7,9,11,12,13,14,15]. We suspected that these contradictory findings are the result of repeated contact of the surface with water during the goniometric measurements. Similar to how the slowed reaction rate of these LIPSS-decorated surfaces allowed for hydrophobic carbonaceous compounds to form on the *boiling-water* preparations over the 30 days following treatment. The increased time required to form the hydrophobic carbonaceous surface chemistry allowed for the water to form some hydrophilic iron oxide on the surface as contact angles were measured. What is presented instead of an exponential growth is a linear increase in water contact angles over the 30 days following the post-processing step. Further, while we may not expect the nearly 150° sessile water contact angles reported by others on their hierarchically-roughened laser micromachined surfaces, we would expect to obtain decidedly hydrophobic surfaces from the *no-post* preparations after 30 days.

Further, the results presented in Figure 4a seem to contradict the hypothesis that the hydrophobic carbonaceous layer is the result of dissociative adsorption of CO_2_ onto the irradiated surface. If that were the case, the 48-h residence in the elevated temperature/pressure CO_2_ reactor should have resulted in *CO_2_-reactor* surfaces with higher hydrophobicity than the *no-post* surfaces, based on previously reported findings [6,13,17]. From Figure 4a, it would appear that the hypothesis of adsorbed hydrocarbons being the source of the hydrophobic carbonaceous surface chemistry is the more likely explanation [12,13,15,18,19]. Whereas both the *no-post* and *CO_2_-reactor* surface present with equivalent average contact contact angles after their residences in lab air. However, as mentioned above, we suspected that these wettability results were being affected by the goniometric measurements themselves.

**Figure 5:**
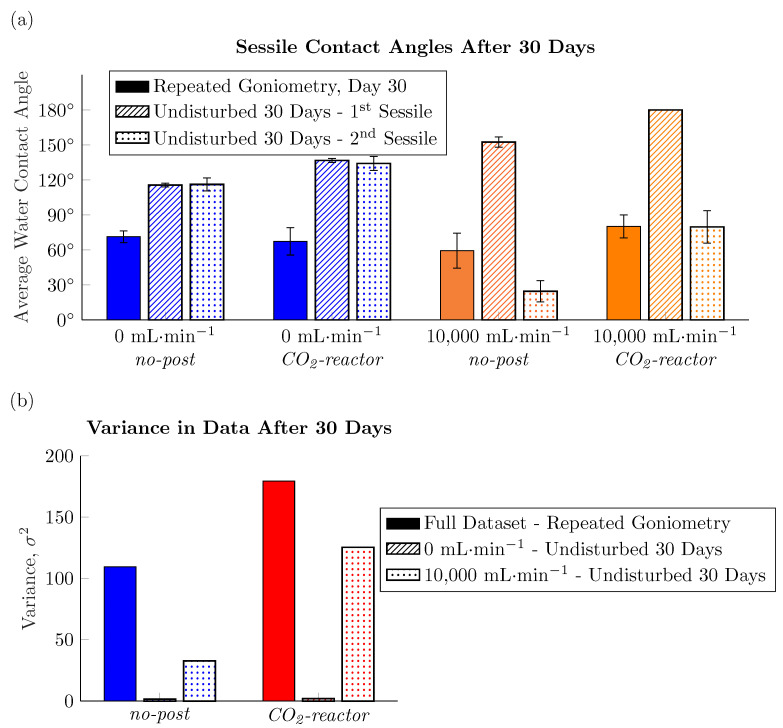
(**a**) Average sessile contact angle measured on *no-post* and *CO_2_-reactor* surfaces left undisturbed for 30 days after fabrication before goniometric measurements were taken. (**b**) Variance in the sessile water contact angle data presented in part (**a**).

This hypothesis of the water contact angle measurements affecting the final wettability of the surfaces was tested by preparing four more LIPSS-decorated samples: (i) 0 mL·min^−1^*no-post*; (ii) 0 mL·min^−1^*CO_2_-reactor*; (iii) 10,000 mL·min^−1^*no-post*; and (iv) 10,000 mL·min^−1^*CO_2_-reactor* and only taking goniometric measurements on their surfaces after 30 days. Figure 5a compares the average water contact angle measured at day 30 on these “undisturbed” preparations with the day 30 measurement taken on those surfaces prepared the same way, but repeatedly tested on the goniometer (i.e., every 10 days for 30 days). It is immediately evident that the average sessile contact angle measured on any of the four undisturbed surfaces is much greater than the average sessile contact angle measured on the same preparation which has come into repeated contact with water over the 30 days of experimentation. In the case of the *no-post* surfaces fabricated with no CO_2_ stream during the micromachining step, the average static contact angle has increased from 71±5° to 116±2° by avoiding any contact with water for the 30 days following fabrication. The hydrophobic sessile contact angle manifested by the undisturbed surface matches well what is reported in the literature for LIPSS-decorated metals [9,15,23,24].

It is interesting to note that Figure 5a now shows that residence in the warm, elevated pressure CO_2_ reactor positively affects the hydrophobicity of the laser micromachined metal sample. Once the 0 mL·min^−1^*CO_2_-reactor* sample is left undisturbed for 30 days following its fabrication, it matches the hypothesis that the dissociative absorption of CO_2_ is (at least partly) responsible for the hydrophobic carbonaceous surface chemistry found on laser micromachined metals. Whereas the average contact angle of the first sessile drop to touch the undisturbed *CO_2_-reactor* surface prepared with no CO_2_ stream during irradiation is 137±2° compared to 116±2° for the *no-post* preparation (and compared to 67±12° for the *CO_2_-reactor* surface with repeated water contact). These drastic changes in the wettability of the surfaces with avoidance of water contact for the 30 days following their fabrication confirms our hypothesis. What is remarkable is just how affected the hydrophobicity of the nascent surface chemistry is by the relatively short contact time with water during the goniometric measurements at 10-day intervals. Micromachining with low fluences, such as in the present work, evidently requires added consideration of avoiding any water contact before the hydrophobic carbonaceous chemical layer forms on the laser irradiated metal, if one desires a highly hydrophobic surface.

The results presented in Figure 5a are also a good recall to our discussion in Section 3.1. Similarly to the surfaces prepared with no streaming CO_2_ during the micromachining step, avoiding contact with water for the first 30 days after fabrication has yielded much higher water contact angles for the first sessile drop placed on the surfaces prepared with CO_2_ flowing at 10,000 mL·min^−1^. In fact, the *CO_2_-reactor* surface, prepared with a 10,000 mL·min^−1^ gas stream during micromachining and no water contact for the first 30 days after fabrication, presented as so hydrophobic that a droplet could not be placed on the surface at all. A video showing a water droplet immediately rolling off the surface is included as Supplementary Materials. Where the behaviours of these undisturbed surfaces diverge is in their wetting performance against the second sessile droplet placed on them. The two samples prepared without the flow of CO_2_ gas during the micromachining steps present as equally hydrophobic to the second sessile drop as to the first sessile drop to touch the surface. On the contrary, the two samples created with a high flow rate of CO_2_ gas present with a much lower average static contact angle with the second sessile drops to touch their surfaces. The blanketing of the surface with non-sintered nanoparticles and agglomerated nanoparticle flakes when micromachining in the presence of the high flow rate CO_2_ stream has led to the same result on the undisturbed surfaces as the surfaces repeatedly tested on the goniometer, as described in Section 3.1. That is, the hydrophobicity of the non-sintered nanoparticles themselves is what imparts the Cassie state non-wetting behaviour of the surface against the first sessile droplet. These hydrophobic nanoparticles/agglomerates are removed with the first sessile drop, exposing the LIPSS for contact with subsequent water.

What is especially interesting to note in Figure 5a is that even after 30 days of aging in the lab air with no water contact, the LIPSS themselves which decorate the *no-post* sample prepared with a high flow rate of CO_2_ gas during micromachining remain hydrophilic. In fact, the second sessile water droplet to touch this undisturbed surface—the first to touch the LIPSS themselves—presents with an average contact angle of 25±9°, statistically equivalent to the average sessile contact angle measured at day 0 on the same surface preparation (see Figure 2a). Figure 3 shows LIPSS visible underneath the nanoparticle agglomerates which homogeneously cover the surface when CO_2_ gas is streamed during micromachining. However, we hypothesize that the chemical reaction at the surface is blocked from occuring by a coverage of small non-sintered nanoparticle clusters. Crystallographic techniques could be used in future studies to distinguish the laser-induced nanoripples, integral to the surface, from non-sintered nanoparticles sitting atop them. Figure 5a shows that even the elevated pressure, temperature, and carbon dioxide-rich environment used to prepare the 10,000 mL·min^−1^*CO_2_-reactor* sample was not sufficient to completely overcome the blocking of the reactive surface by nanoparticles and nanoparticle agglomerates. Even though this *CO_2_-reactor* sample is more hydrophobic than the *no-post* sample prepared with the same 10,000 mL·min^−1^ CO_2_ stream during the micromachining step, both the high flow rate surfaces are much less hydrophobic than the two undisturbed samples prepared with no gas stream during irradiation.

The two major observations made in this work with regards to fabricating LIPSS-decorated surfaces with tuned wettability bear repeating. The first is that any non-sintered nanoparticles and nanoparticle agglomerates should be removed from the surface prior to any post processing steps to completely expose the reactive surface. However, it should be noted that removal of non-sintered nanoparticles is accomplished by ultrasonication, which exposes the reactive irradiated surface to the solvent itself. This has recently been shown to also affect the final wettability of laser micromachined metals [25]. Other techniques such as laser cleaning with photoacoustic monitoring may also prove effective in removing non-sintered nanoparticle agglomerates from laser-irradiated surfaces [26]. If non-sintered nanoparticle agglomerates are not removed, then the wettability measured is not that of the LIPSS themselves, but rather the compound LIPSS-nanoparticle agglomerate system, which inherently lacks robustness. The second observation is that the reformation of the surface chemistry on a LIPSS-only surface is much slower than hierarchically-roughened laser irradiated surfaces due to the lowered fluence employed for micromachining. As such, care must be taken to not disturb the nascent surface chemistry before it has sufficiently reacted to form either a robust hydrophilic oxide layer or a robust hydrophobic carbonaceous layer. For the fabrication of a hydrophilic surface, this means that the freshly irradiated substrate must be kept in a boiling water bath for longer than the two days reported necessary for metals irradiated with higher fluences [6]; For the fabrication of a hydrophobic surface, this means that the irradiated substrate must be kept away from even brief contact with water. The high level of homogeneity that is achieved when both recommendations are followed is exemplified by the very small variances in the contact angle data measured on the undisturbed surfaces. Figure 5b presents the variance in the sessile contact angles measured on those surfaces prepared with no stream of gas flowing during the micromachining step (i.e., low amount of non-sintered nanoparticles/agglomerates on the surface) and left undisturbed by water contact for the first 30 days following fabrication. The comparison with the contrary surface preparations is staggering. Both repeated contact with water through goniometric measurements and failure to remove non-sintered nanoparticles/agglomerates results in much more heterogeneous surfaces.

## 4. Conclusions

In this work, we have taken a systematic look at the effect of surface handling in the early times after irradiation on the final wettability of stainless-steel decorated with laser-induced periodic surface structures. In doing so we have been able to rigorously demonstrate that exposure to a CO_2_-rich environment after micromachining does, in fact, lead to increased hydrophobicity. This proves what we have empirically known from our lab’s work with laser irradiated metals: that CO_2_ as well as airborne hydrocarbons can be the source of the hydrophobic carbonaceous layer which forms in the weeks following micromachining.

While performing this work, we have made a few interesting observations which we see as pertinent to metal laser micromachining getting transferred to industrial applications. The first observation is that the waste product of ultrafast laser micromachining—nanoparticles and nanoparticle agglomerates—can affect the wettability of the surface. We have shown that as these non-sintered particles sit on top of the irradiated surface, they become hydrophobic with residence in a CO_2_-rich environment (or indeed with aging in lab air), while blocking this reaction from proceeding on the irradiated surface itself. Hence, contact angle measurements taken on such a surface will be measuring the wettability of the water-nanoparticle (agglomerate) system, and not the wettability of the actual micromachined metal surface. The substrate will behave much differently to subsequent contact with water as hydrophobic nanoparticle (agglomerates) are carried away, exposing the underlying hydrophillic micromachined surface. The second pertinent observation is that the metal surfaces prepared in the present study to possess only laser-induced periodic surface structures underwent much slower surface chemical reactions than those previously reported on metals hierarchically roughened by laser micromachining. This point is important with regards to industrial scale-up as we have shown that fabrication of a stable hydrophilic LIPSS-decorated stainless-steel requires a longer residence in boiling water after irradiation than a hierarchically-roughened substrate. Similarly, fabrication of a stable hydrophobic LIPSS-decorated stainless-steel requires that water contact be avoided at early times while a carbonaceous layer forms on the surface.

The observations and recommendations made in the present work can hopefully help guide the handling procedures for laser irradiated metal substrates in the lab and industry alike. Taking into account the need to remove non-sintered nanoparticles and nanoparticle agglomerates as well as requisite surface reaction time requirements can greatly aid in achieving desired wettabilities on laser micromachined metals.

## Figures and Tables

**Table 1:** ASTM A751/14a chemical analysis of 316 stainless-steel substrate.

C%	Cr%	Cu%	Mn%	Mo%	N%	Ni%	P%	S%	Si%
0.0205	16.5820	0.4420	1.1830	2.0205	0.0465	10.0700	0.0310	0.0010	0.2750

## Data Availability

The data presented in this study are available on request from the corresponding author.

## Supplementary Materials

The following are available online at https://www.mdpi.com/article/10.3390/nano11040973/s1, Figure S1: Effect of CO_2_ flow rate on advancing contact angles, Figure S2: Effect of post-processing on advancing contact angles, Video S1: Droplet_Rolloff.
