# Supplementary material for: The Tuning of LIPSS Wettability during Laser Machining and through Post-Processing
Source: Nanomaterials (Basel). 2021 Apr 10;11(4):973. doi: 10.3390/nano11040973 (PMC8069829; doi:10.3390/nano11040973)
Supplement: Supplementary file 1 [file nanomaterials-11-00973-s001.zip › Supplementary/LIPSS_Wettability_Tuning_Manuscript_Supplementary.pdf]

The tuning of LIPSS wettability through manipulation of the irradiation atmosphere and post-processing carbonaceous layer growth

Supplementary Materials

Michael J. Wood, Phillip Servio, Anne-Marie Kietzig*

McGill University Department of Chemical Engineering,
3610 University St., Montréal, Québec, H3A 0C5, Canada

*Corresponding Author: anne.kietzig@mcgill.ca

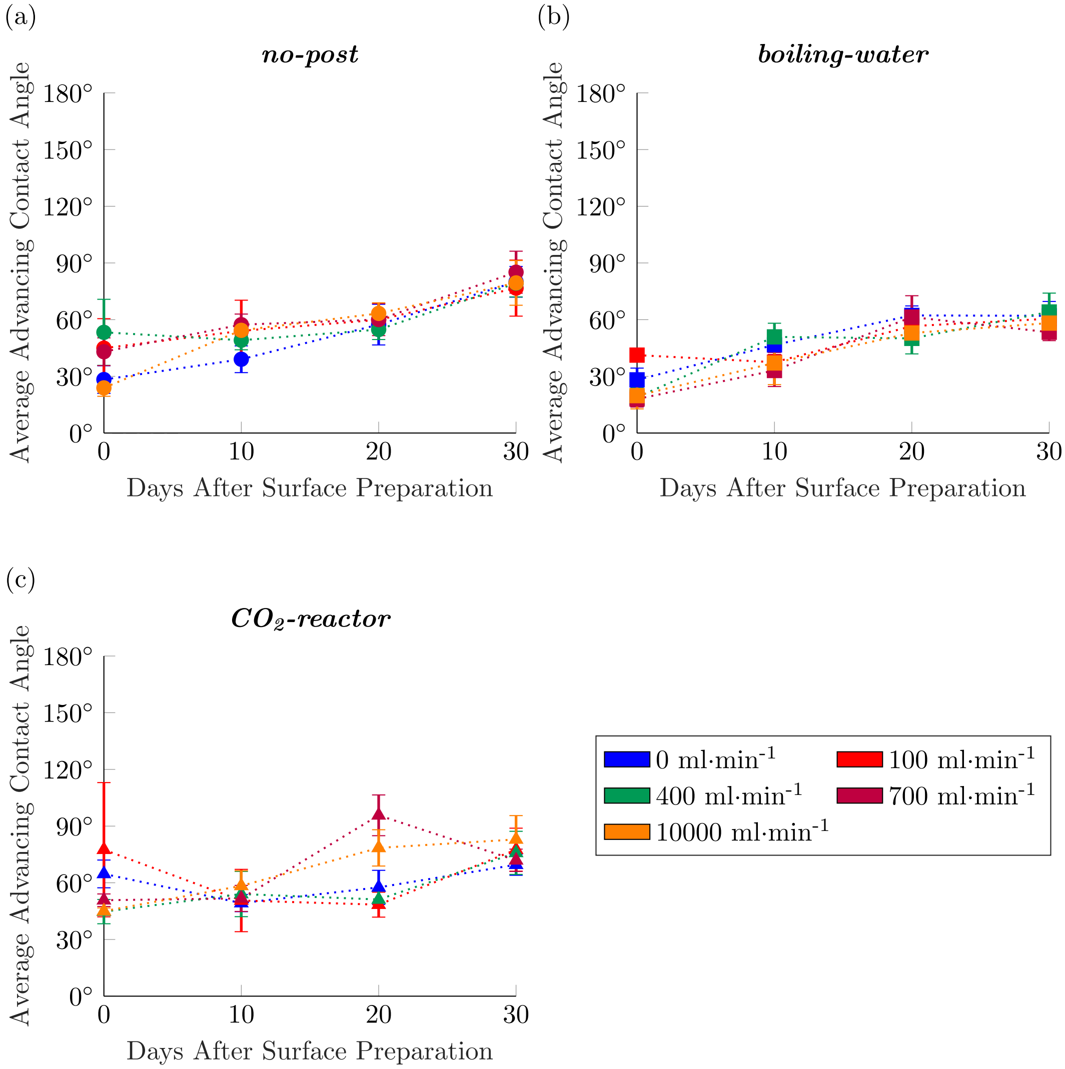

Figure S1: Average advancing water contact angle measurements taken over the 30 days following laser micromachining/post-processing on surfaces prepared with: (**a**) no post-processing step; (**b**) 48 hours in boiling water; and (**c**) 48 hours in the CO_2 reactor.

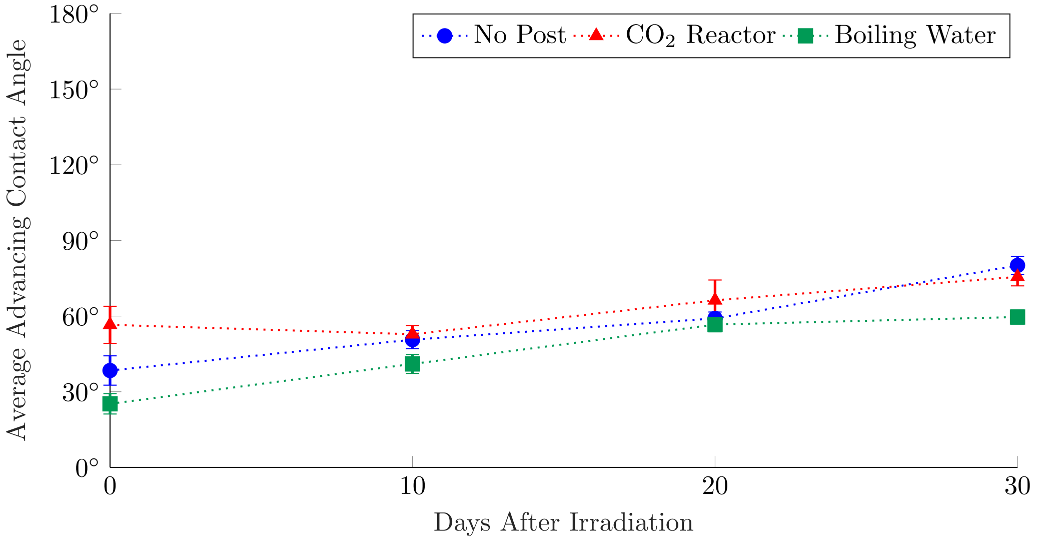

Figure S2: Average advancing contact angle measured on the *no-post*, *CO_2-reactor*, and *boiling-water* surfaces regardless of CO_2 jet volumetric flow rate during micromachining over the 30 days following surface preparation.
